# Supplementary material for: Development of a core outcome set for congenital pulmonary airway malformations: study protocol of an international Delphi survey
Source: BMJ Open. 2021 Apr 12;11(4):e044544. doi: 10.1136/bmjopen-2020-044544 (PMC8047990; doi:10.1136/bmjopen-2020-044544)
Supplement: Author's manuscript [file bmjopen-2020-044544.draft_revisions.pdf]

BMJ Open is committed to open peer review. As part of this commitment we make the peer review history of every article we publish publicly available.

When an article is published we post the peer reviewers' comments and the authors' responses online. We also post the versions of the paper that were used during peer review. These are the versions that the peer review comments apply to.

The versions of the paper that follow are the versions that were submitted during the peer review process. They are not the versions of record or the final published versions. They should not be cited or distributed as the published version of this manuscript.

BMJ Open is an open access journal and the full, final, typeset and author-corrected version of record of the manuscript is available on our site with no access controls, subscription charges or pay-per-view fees (http://bmjopen.bmj.com).

If you have any questions on BMJ Open's open peer review process please email info.bmjopen@bmj.com

Development of a Core Outcome Set for Congenital Pulmonary Airway Malformations: study protocol of an international Delphi survey

Journal:	*BMJ Open*
Manuscript ID	bmjopen-2020-044544
Article Type:	Protocol
Date Submitted by the Author:	09-Sep-2020

Complete List of Authors:	Hermelijn, Sergei; Erasmus MC Sophia Children Hospital, Pediatric Surgery<br>Kersten, Casper; Erasmus MC Sophia Children Hospital, Pediatric Surgery<br>Mullassery, Dhanya; Great Ormond Street Hospital for Children<br>Cobanoglu, Nazan; Ankara University Faculty of Medicine<br>Gartner, Silvia; Vall d'Hebron Hospital Universitari<br>Bagolan, Pietro; Ospedale Pediatrico Bambino Gesu, Department of Medical and Surgical Neonatology<br>Mesas Burgos, Carmen; Karolinska Institutet<br>Sgro, Alberto; Padua University Hospital<br>Heyman, Stijn; ZNA-GZA Paola Children's Hospital<br>Till, Holger; Medical University of Graz<br>Suominen, Janne; University of Helsinki Children's Hospital<br>Schurink, Maarten; Radboud University Medical Centre Amalia Children's Hospital<br>Desender, Liesbeth; Ghent University Faculty of Medicine and Health Sciences<br>Losty, Paul; University of Liverpool,<br>Ertresvag, Kjetil; Oslo University Hospital<br>Tiddens, Harm A. W. M.; Erasmus MC Sophia Children Hospital, Pediatric Pulmonology<br>Wijnen, Rene; Erasmus University Medical Center - Sophia Children's Hospital, Paediatric Surgery<br>Schnater, Marco; Erasmus Medical Center, Paediatric Surgery

Keywords:	Paediatric intensive & critical care < ANAESTHETICS, NEONATOLOGY, Paediatric thoracic medicine < PAEDIATRICS, PAEDIATRIC SURGERY, Paediatric thoracic surgery < PAEDIATRIC SURGERY

SCHOLARONE™ Manuscripts

I, the Submitting Author has the right to grant and does grant on behalf of all authors of the Work (as defined in the below author licence), an exclusive licence and/or a non-exclusive licence for contributions from authors who are: i) UK Crown employees; ii) where BMJ has agreed a CC-BY licence shall apply, and/or iii) in accordance with the terms applicable for US Federal Government officers or employees acting as part of their official duties; on a worldwide, perpetual, irrevocable, royalty-free basis to BMJ Publishing Group Ltd ("BMJ") its licensees and where the relevant Journal is co-owned by BMJ to the co-owners of the Journal, to publish the Work in this journal and any other BMJ products and to exploit all rights, as set out in our licence.

The Submitting Author accepts and understands that any supply made under these terms is made by BMJ to the Submitting Author unless you are acting as an employee on behalf of your employer or a postgraduate student of an affiliated institution which is paying any applicable article publishing charge ("APC") for Open Access articles. Where the Submitting Author wishes to make the Work available on an Open Access basis (and intends to pay the relevant APC), the terms of reuse of such Open Access shall be governed by a Creative Commons licence – details of these licences and which Creative Commons licence will apply to this Work are set out in our licence referred to above.

Other than as permitted in any relevant BMJ Author's Self Archiving Policies, I confirm this Work has not been accepted for publication elsewhere, is not being considered for publication elsewhere and does not duplicate material already published. I confirm all authors consent to publication of this Work and authorise the granting of this licence.

For peer review only

Development of a Core Outcome Set for Congenital Pulmonary Airway Malformations: study protocol of an international Delphi survey

S.M. Hermelijn [1], C.M. Kersten [1], D. Mullasery [2], N. Cobanoglu [3], S. Gartner [4], P. Bagolan [5], C. Mesas Burgos [6], A. Sgrò [7], S. Heyman [8], H. Till [9], J. Suominen [10], M. Schurink [11], L. Desender [12], P.D. Losty [13], K. Ertresvag [14], H.A.W.M. Tiddens [15], R.M.H. Wijnen [1], J.M. Schnater [1] on behalf of the CONNECT study consortium COS development group

[1] Department of Paediatric Surgery, Erasmus Medical Centre-Sophia Children's Hospital, Rotterdam, the Netherlands
[2] Department of Paediatric Surgery, Great Ormond Street Hospital, London, United Kingdom
[3] Department of Paediatric Pulmonology, Ankara University, School of Medicine, Ankara, Turkey
[4] Department of Paediatric Pulmonology, Hospital Universitari Vall d´Hebron, Barcelona, Spain
[5] Department of Paediatric Surgery, Bambino Gesù Pediatric Hospital,Rome, Italy
[6] Department of Paediatric Surgery, Karolinska Institutet, Stockholm, Sweden
[7] Department of Paediatric Surgery, University Hospital Padua, Padua, Italy
[8] Department of Paediatric Surgery, ZNA-GZA Paola Children's Hospital, Antwerp, Belgium
[9] Department of Paediatric Surgery, Medical University of Graz, Graz, Austria
[10] Department of Paediatric Surgery, Helsinki Children's Hospital, Helsinki, Finland
[11] Department of Paediatric Surgery, Radboud University Medical Centre-Amalia Children's Hospital, Nijmegen, the Netherlands
[12] Department of Paediatric Surgery, Ghent University Hospital, Ghent, Belgium
[13] Department of Paediatric Surgery, Alder Hey Children's Hospital, Liverpool, United Kingdom
[14] Department of Paediatric Surgery, Oslo University Hospital, Oslo, Norway
[15] Department of Paediatric Surgery, Erasmus Medical Centre-Sophia Children's Hospital, Rotterdam, the Netherlands

Corresponding Author:
J.M. Schnater, MD, PhD
Department of Paediatric Surgery, Erasmus MC – Sophia Children's Hospital
Postal address: Postbus 2060. 3000 CB Rotterdam
Email: j.schnater@erasmusmc.nl
Telephone number: (+31)107036214

Word count: 1776 words

For peer review only

Abstract

Introduction

A worldwide lack of consensus exists on the optimal management of asymptomatic Congenital Pulmonary Airway Malformation (CPAM) even though the incidence is increasing. Either a surgical resection is performed or a wait-and-see policy is employed, depending on the treating physician. Management is largely based on expert-opinion and scientific evidence is scarce. A large variety in outcome measures is seen between studies making comparison difficult and highlighting the lack of consensus in outcome measures as well. We aim to define a core outcome set which includes the most important core outcome parameters for paediatric patients with an asymptomatic CPAM.

Methods and analysis

This study will include a critical appraisal of the current literature followed by a three-stage Delphi process with two stakeholder groups. One surgical group including paediatric as well as thoracic surgeons, and a non-surgeon group including paediatric pulmonologists, intensive care and neonatal specialists. All participants will score outcome parameters according to their level of importance and the most important parameters will be determined by consensus.

Ethics and Dissemination

The core outcome set development is part of a larger project which is aimed at determining the optimal management of asymptomatic CPAM patients. This will be an international randomized controlled trial: The CONNECT (COllaborative Neonatal NEtwork for the first CPAM Trial).

Strength and limitations of this study

- No consensus exists on the optimal management of patients with an asymptomatic Congenital Pulmonary Airway Malformation (CPAM).
- Prospective studies on postnatal management are lacking, and cohort studies vary widely in end terms.
- Optimal timing and modality to monitor asymptomatic patients are unknown.
- Core outcome sets are a disease-specific collection of the most important outcomes that have been identified by consensus between key stakeholders.
- This protocol describes an international online Delphi survey aimed at identifying the most important core outcome parameters for paediatric patients with an asymptomatic CPAM.

Keywords: congenital lung disease, lung malformation, pediatric pulmonology, Congenital cystic adenomatoid malformation, Congenital pulmonary airway malformation

For peer review only

Introduction

Congenital Pulmonary Airway Malformation (CPAM), formerly known as Congenital Cystic Adenomatoid Malformation (CCAM), is the most common congenital lung abnormality (CLA) [1]. The incidence of CPAM has increased up to 4 per 10,000 births over the last years [2]. Most infants are asymptomatic but others may show symptoms such as neonatal respiratory distress, persistent cough or recurrent lung infections in the first years of life [3]. A worldwide lack of consensus exists on the optimal management and follow-up of infants with an asymptomatic CPAM [4 5]. Prospective studies on postnatal management are lacking, and cohort studies vary widely in end terms [2 6].

An asymptomatic CPAM is either surgically resected or a wait-and-see policy is employed following local guidelines. Either way, cases are ideally discussed in a multidisciplinary team in which parental wish is taken into account as well. To date, no definitive evidence exists on the optimal management and factors for predicting symptoms are still being investigated [6]. The arguments for a wait-and-see policy are that the malformation is originally benign, potentially regresses, and in most cases remains asymptomatic [7]. The arguments for surgical management include the risk of recurrent lung infections - which could make surgery more difficult-, risk for acute respiratory distress, potential malignant degeneration, parental anxiety, and allowing for compensatory lung growth [8 9].

Consensus needs to be reached on the outcome measures and their timing that could be applied in international studies aimed at identifying the optimal management of asymptomatic CPAM. A core outcome set (COS) is a disease-specific collection of outcomes that have been identified by consensus between key stakeholders as being the most important in determining success of a treatment [10]. Such consensus is often reached through a Delphi method in which stakeholders anonymously rate outcome measures according to their importance, in one or multiple rounds [11]. We aim to develop a COS for CPAM patients using the Delphi method as a tool for reaching consensus and present the study protocol for this.

Methods

The COS development will follow the Core Outcome Set-STAndards for Development (COS-STAD) Recommendations [12] and the Core Outcome Measures in Effectiveness Trials (COMET) handbook [11]. This COS development study was registered with the COMET Initiative in May 2020 (http://www.comet-initiative.org/studies/details/1570).

Scope

The COS to be developed is intended for use in a randomized controlled trial: The CONNECT (COllaborative Neonatal NEtwork for the first CPAM Trial). This trial will be performed by the CONNECT study consortium and aims to identify the optimal management of asymptomatic CPAM patients. The COS is intended for pediatric patients up until the age of 18 years and will not include fetal outcome. Outcome parameters as well as their measurement instruments, and age at assessment will be included.

Study design

The COS will be developed in an online, three-round, Delphi process, preceded by an appraisal of previously published literature.

Literature review

A systematic literature review is recommended to yield an initial outcome set for the first round in the Delphi process [11]. To date, no randomized studies have yet been done and studies report a large variety in outcome parameters [6]. We will research two recent extensive literature reviews, published in a special issue of a pediatric surgery scientific journal, each covering opposing thoughts on the arguments for surgical management [8] or a wait-and-see policy [7]. In addition, we will scrutinize a

recent systematic review and meta-analysis which covers the risks associated with either surgical resection or a wait-and-see policy of asymptomatic CPAM [3]. All relevant outcome parameters identified from the above-mentioned reviews will be used in the first round of the Delphi process.

Stakeholders and recruitment

Pediatric surgeons and thoracic surgeons are the health care professionals who are most frequently involved in the operative management of CPAM patients. Initial consultation or follow-up is variably done by pediatric surgeons, maternal-fetal medicine specialists, neonatologists, pediatricians or pediatric pulmonologists. We therefore decided to form two stakeholder groups: (1) Surgeons (e.i. pediatric and thoracic surgeons); and (2) Non-surgeons (e.g. maternal-fetal medicine specialists, pediatricians, pediatric pulmonologists and neonatologists).

We will recruit study participants through an existing international network of pediatric surgeons and pulmonologists whom have expressed interest in collaborating on the CONNECT trial. We will inform potential participants on the aims and procedures of the Delphi process and encourage them to enroll other specialists involved in the care of CPAM patients in their own centers. Prior to the first round, those who have been found willing to participate will be sent an email explaining the aims and procedures of the Delphi process, and emphasizing the importance of finishing each round within the allocated time.

Sample size

There is no consensus on the optimal sample size for a Delphi study [11]; recruitment will therefore be based on the prospective study for which it is primarily intended (CONNECT). For the CONNECT trial, we aim to include at least 12 international centres, and therefore set the minimum of participants in each stakeholder group in the final round at 12. To reduce bias, no more than two participants from a single centre can participate in a stakeholder group. To minimize attrition bias in consecutive rounds, we aim to achieve that 75% of participants complete a round [11 13]. Therefore, the minimum number of participants in each stakeholder group for round 1 will be 21, and 16 for round 2. We believe that these minimum numbers constitute a representative sample, considering the rarity of the disease and the limited number of professionals with experience in managing this disease. Previous Delphi COS studies in paediatric surgery used similar numbers for investigating a more common disease such as appendicitis [14 15].

Attrition bias

Attrition bias will be assessed separately for each round of the Delphi process, and separately in each stakeholder group. In each group, the median score of every outcome will be compared using the Wilcoxon rank-sum test, between those only completing the previous round and those completing the consecutive round as well [11].

Delphi study

Consensus

Participants will be asked to score each outcome parameter using the Grading of Recommendations, Assessment, Development and Evaluations (GRADE) scale [16]. The 9-point Likert scale will label 1–3 as “not important”, 4–6 as “important but not critical” and 7–9 as “critical”. Consensus for inclusion is reached if ≥70% of participants rate the outcome parameter 7–9 and <15% rate it 1–3. Consensus for exclusion is reached if >70% participants rate the outcome 1–3 and <15% rate it 7–9. Outcomes not meeting these definitions will be classified as ‘no consensus’ [11].

Timeline

Participants will be asked to complete each round of the Delphi process within 4 weeks. A weekly reminder email will be sent to those who then have not yet completed the survey. Those failing to

complete the questionnaire within the allocated 4 weeks will be excluded from next rounds. The deadline shall be extended if the projected minimum sample size has not been reached and those failing to complete the questionnaire shall be approached individually.

Delphi round 1

The three-round Delphi process shall be performed using "Welphi", an online data system specifically developed for this use [17]. All participants shall be approached simultaneously and asked to rate each of the previously identified outcome parameters on importance as follows: *" How important would you rate the following outcome parameter including measurement instrument and age in determining the best management of asymptomatic CPAM patients? "*.

Participants are invited to suggest additional outcome parameters stating: (1) the outcome parameter, (2) the measurement instrument, and (3) the age at assessment. These additional outcome parameters will be scored in the second round.

Delphi round 1 analysis

Outcome parameters will be analyzed separately for each stakeholder group (surgeons and non-surgeons) and all parameters will be included in the second round of the Delphi process. The additional outcomes provided by participants will be reviewed to confirm they represent new outcomes. If confirmed, the item in question will be included in the second round as well.

Delphi round 2

All participants who completed the first round will automatically be invited to participate in round 2. Per stakeholder group, the median scores assigned in the first round will be made known. This will allow participants to consider the views of the other participants in the stakeholder group. They will be invited to look at all items again, and consider adjusting their own scores. Furthermore, they will be asked to score the newly added outcome parameters suggested in the first round.

Delphi round 2 analysis

All outcome parameters meeting consensus criteria for exclusion by all participants will be excluded from the third round. All other parameters will be included in the third round.

Delphi round 3

All participants who completed the first and second rounds will be invited to participate in the third round. The median score of their own stakeholder group and the score of the other stakeholder group will be presented to participants. This will allow participants to consider the views of the other stakeholder group before rescoring the outcomes. They will be invited to look at all remaining items again, and consider adjusting their own scores. In addition, participants will be asked to identify a single outcome parameter which is the most important for determining the treatment choice in asymptomatic CPAM patients according to them.

Delphi round 3 analysis and final COS development

All outcome parameters meeting the criteria for consensus of inclusion by all participants, will be included in the final core outcome set. All other outcome parameters will be excluded. To achieve a COS which is feasible for clinical use in trials, we aim to include a maximum of 10 outcome parameters in the final COS. If the number of outcome parameters meeting the criteria for consensus of inclusion greatly exceeds this maximum number, we will only include the 10 outcomes with the highest level of consensus for the COS and report those excluded in this stage. The level of consensus will be determined by the median score of each outcome parameter in round 3.

The final COS will be annotated according to the outcome taxonomy which was constructed to maximize future data harmonization . Additionally, the final COS will be divided into the four core

For peer review only - http://bmjopen.bmj.com/site/about/guidelines.xhtml

areas of the OMERACT filter: death, life impact, pathophysiological manifestations, and resource use [10].

Ethics and dissemination

Electronic informed consent will be obtained from all participants. The final COS will be published in an international peer-reviewed scientific journal and on the COMET Initiative website (http://www. comet- initiative.org).

Data collection and confidentiality

Participants will complete questionnaires using the “Welphi” survey tool [17]. Anonymized data will be stored on a secure online server and will be managed according to the European General Data Protection Regulation [18].

For peer review only

Contributors

All authors contributed to the design of this protocol. SH, DM, NC, SG, PB, CMB, AS, SH, JS, MS, LD, KE, HAT, RW, JMS initiated the project. The protocol was drafted by SH and CK. The protocol was critically reviewed by DM, NC, SG, PB, CMB, AS, SH, HT, JS, MS, LD, PL, KE, HAT, RW, JMS. All authors contributed to the manuscript and read and approved the final manuscript. The CONNECT study consortium COS development group consists of all participants of the Delphi process. They have all read, refined and approved the final manuscript.

Funding statement

The authors have not received a specific grant for this research from any funding agency in the public, commercial or non-profit sectors.

Competing interests

None declared.

Patient and public involvement

No patient involved

Provenance and peer review

Not commissioned; externally peer reviewed.

Collaborators: The following people form the CONNECT study consortium COS development group: S.M. Hermelijn, C.M. Kersten, J. Schnater, R.M.H. Wijnen, H.A.W.M. Tiddens, S.C.M. Cochius - den Otter (Sophia Children's Hospital, Rotterdam-the Netherlands); J. Suominen, M. Pakarinen, L. Martelius (Helsinki Children's Hospital, Helsinki-Finland); S. Heyman, D. Vervloessem (ZNA-GZA Paola Children's Hospital, Antwerp-Belgium); H. Steyaert (Queen Fabiola Children's University Hospital, Brussels-Belgium); A. Sgrò, P. Gamba (University Hospital Padua, Padua-Italy); M. Schurink, S. van der Heide, J. Roukema (Amalia Children's Hospital, Nijmegen-the Netherlands); N. Rikkers-Mutsaerts (Leiden University Medical Centre, Leiden-the Netherlands); S. Terheggen-Lagro, S. de Beer, E. Haarman (Amsterdam University Medical Centre, Amsterdam-the Netherlands); H. Till, G. Singer (Medical University of Graz, Graz-Austria); M. Metzelder, P Sezen (Medizinische Universität Wien, Vienna-Austria); L. Desender, H. Schaballie (Ghent University Hospital, Ghent-Belgium); N. Cobanoglu, G. Gollu (Ankara University, School of Medicine, Ankara-Turkey); M. Stanton (University Hospital Southampton, Southampton-United Kingdom); A. Bonnard (Hopital Universitaire Robert Debré, Paris-France); R. Sfeir (Hopital Jeanne de Flandre, Lille-France); N. Muthialu, D. Mullassery, C. Wallis (Great Ormond Street Hospital, London-United Kingdom); D. Cox (Children's Health Ireland at Crumlin, Dublin-Ireland); Pietro Bagolan, F. Morini (Bambino Gesù Pediatric Hospital, Rome-Italy); C. Mesas Burgos, P. Conner, E. Caffrey Osvald, C. Bitkover (Karolinska Institutet, Stockholm-Sweden); H. Decaluwé, M. Proesmans, M. Boon, J. Deprest (University Hospital Leuven, Leuven-Belgium); S. Gartner, A. Lain (Hospital Universitari Vall d´Hebron, Barcelona-Spain); P. Losty, Ian Sinha (Alder Hey Children's Hospital, Liverpool-United Kingdom); I. Yardley (Evelina London Children's Hospital, London-United Kingdom); M. Singh (Birmingham Children's Hospital, Birmingham-United Kingdom); L. Wessel, K. Zahn, T. Schaible (University Hospital Mannheim, Mannheim-Germany); N. Qvist (Odense Univeristy Hospital, Odense-Denmark); M. Zampoli (Red Cross Children's Hospital, Cape town-South Africa); G. Aksnes (Oslo University Hospital, Oslo-Norway); C. K. van der Ent, K.M. Winter-de Groot (University Medical Centre Utrecht, Utrecht-the Netherlands); R. Peters (Royal Manchester Children's Hospital, Manchester-United Kingdom); E. Hannon (Leeds Children's Hospital, Leeds-United Kingdom); Q. Jöbsis, M. Bannier (Maastricht University Medical Centre, Maastricht-the Netherlands).

For peer review only

References

1. EUROCAT. Prevalence tables. 2007-2018. http://www.eurocat-network.eu/ACCESSPREVALENCEDATA/PrevalenceTables (accessed Jan 2019).
2. Stocker LJ, Wellesley DG, Stanton MP, et al. The increasing incidence of foetal echogenic congenital lung malformations: an observational study. Prenat Diagn 2015;**35**(2):148-53.
3. Stanton M, Njere I, Ade-Ajayi N, et al. Systematic review and meta-analysis of the postnatal management of congenital cystic lung lesions. J Pediatr Surg 2009;**44**(5):1027-33.
4. Lo AY, Jones S. Lack of consensus among Canadian pediatric surgeons regarding the management of congenital cystic adenomatoid malformation of the lung. J Pediatr Surg 2008;**43**(5):797-9.
5. Morini F, Zani A, Conforti A, et al. Current Management of Congenital Pulmonary Airway Malformations: A "European Pediatric Surgeons' Association" Survey. Eur J Pediatr Surg 2018;**28**(1):1-5.
6. Wong KKY, Flake AW, Tibboel D, et al. Congenital pulmonary airway malformation: advances and controversies. Lancet Child Adolesc Health 2018;**2**(4):290-97.
7. Stanton M. The argument for a non-operative approach to asymptomatic lung lesions. Semin Pediatr Surg 2015;**24**(4):183-6.
8. Singh R, Davenport M. The argument for operative approach to asymptomatic lung lesions. Semin Pediatr Surg 2015;**24**(4):187-95.
9. Hermelijn SM, Wolf JL, Dorine den Toom T, et al. Early KRAS oncogenic driver mutations in non-mucinous tissue of Congenital Pulmonary Airway Malformations as an indicator of potential malignant behavior. Hum Pathol 2020.
10. Boers M, Kirwan JR, Gossec L, et al. How to choose core outcome measurement sets for clinical trials: OMERACT 11 approves filter 2.0. J Rheumatol 2014;**41**(5):1025-30.
11. Williamson PR, Altman DG, Bagley H, et al. The COMET Handbook: version 1.0. Trials 2017;**18**(Suppl 3):280.
12. Kirkham JJ, Davis K, Altman DG, et al. Core Outcome Set-STAndards for Development: The COS-STAD recommendations. PLoS Med 2017;**14**(11):e1002447.
13. Bruce I, Harman N, Williamson P, et al. The management of Otitis Media with Effusion in children with cleft palate (mOMEnt): a feasibility study and economic evaluation. Health Technol Assess 2015;**19**(68):1-374.
14. Sherratt FC, Eaton S, Walker E, et al. Development of a core outcome set to determine the overall treatment success of acute uncomplicated appendicitis in children: a study protocol. BMJ Paediatr Open 2017;**1**(1):e000151.
15. Knaapen M, Hall NJ, van der Lee JH, et al. Establishing a core outcome set for treatment of uncomplicated appendicitis in children: study protocol for an international Delphi survey. BMJ Open 2019;**9**(5):e028861.
16. Guyatt GH, Oxman AD, Kunz R, et al. GRADE guidelines: 2. Framing the question and deciding on important outcomes. J Clin Epidemiol 2011;**64**(4):395-400.
17. Welphi. www.welphi.com. 2020.
18. de Magalhães ST. The European Union’s General Data Protection Regulation (GDPR).

For peer review only

Development of a Core Outcome Set for Congenital Pulmonary Airway Malformations: study protocol of an international Delphi survey

Journal:	*BMJ Open*
Manuscript ID	bmjopen-2020-044544.R1
Article Type:	Protocol
Date Submitted by the Author:	07-Feb-2021
Complete List of Authors:	Hermelijn, Sergei; Erasmus MC Sophia Children Hospital, Pediatric Surgery<br>Kersten, Casper; Erasmus MC Sophia Children Hospital, Pediatric Surgery<br>Mullassery, Dhanya; Great Ormond Street Hospital for Children<br>Muthialu, Nagarajan; Great Ormond Street Hospital for Children<br>Cobanoglu, Nazan; Ankara University Faculty of Medicine<br>Gartner, Silvia; Vall d'Hebron Hospital Universitari<br>Bagolan, Pietro; Ospedale Pediatrico Bambino Gesu, Department of Medical and Surgical Neonatology<br>Mesas Burgos, Carmen; Karolinska Institutet<br>Sgro, Alberto; Padua University Hospital<br>Heyman, Stijn; ZNA-GZA Paola Children's Hospital<br>Till, Holger; Medical University of Graz<br>Suominen, Janne; University of Helsinki Children's Hospital<br>Schurink, Maarten; Radboud University Medical Centre Amalia Children's Hospital<br>Desender, Liesbeth; Ghent University Faculty of Medicine and Health Sciences<br>Losty, Paul; University of Liverpool,<br>Ertresvag, Kjetil; Oslo University Hospital<br>Tiddens, Harm A. W. M.; Erasmus MC Sophia Children Hospital, Pediatric Pulmonology<br>Wijnen, Rene; Erasmus University Medical Center - Sophia Children's Hospital, Paediatric Surgery<br>Schnater, Marco; Erasmus Medical Center, Paediatric Surgery
**Primary Subject Heading**:	Surgery
Secondary Subject Heading:	Paediatrics, Respiratory medicine, Surgery
Keywords:	Paediatric intensive & critical care < ANAESTHETICS, NEONATOLOGY, Paediatric thoracic medicine < PAEDIATRICS, PAEDIATRIC SURGERY, Paediatric thoracic surgery < PAEDIATRIC SURGERY

I, the Submitting Author has the right to grant and does grant on behalf of all authors of the Work (as defined in the below author licence), an exclusive licence and/or a non-exclusive licence for contributions from authors who are: i) UK Crown employees; ii) where BMJ has agreed a CC-BY licence shall apply, and/or iii) in accordance with the terms applicable for US Federal Government officers or employees acting as part of their official duties; on a worldwide, perpetual, irrevocable, royalty-free basis to BMJ Publishing Group Ltd ("BMJ") its licensees and where the relevant Journal is co-owned by BMJ to the co-owners of the Journal, to publish the Work in this journal and any other BMJ products and to exploit all rights, as set out in our licence.

The Submitting Author accepts and understands that any supply made under these terms is made by BMJ to the Submitting Author unless you are acting as an employee on behalf of your employer or a postgraduate student of an affiliated institution which is paying any applicable article publishing charge ("APC") for Open Access articles. Where the Submitting Author wishes to make the Work available on an Open Access basis (and intends to pay the relevant APC), the terms of reuse of such Open Access shall be governed by a Creative Commons licence – details of these licences and which Creative Commons licence will apply to this Work are set out in our licence referred to above.

Other than as permitted in any relevant BMJ Author's Self Archiving Policies, I confirm this Work has not been accepted for publication elsewhere, is not being considered for publication elsewhere and does not duplicate material already published. I confirm all authors consent to publication of this Work and authorise the granting of this licence.

For peer review only

Development of a Core Outcome Set for Congenital Pulmonary Airway Malformations: study protocol of an international Delphi survey

S.M. Hermelijn [1], C.M. Kersten [1], D. Mullassery [2], N. Muthialu [2], N. Cobanoglu [3], S. Gartner [4], P. Bagolan [5], C. Mesas Burgos [6], A. Sgrò [7], S. Heyman [8], H. Till [9], J. Suominen [10], M. Schurink [11], L. Desender [12], P.D. Losty [13], K. Ertresvag [14], H.A.W.M. Tiddens [15], R.M.H. Wijnen [1], J.M. Schnater [1] on behalf of the CONNECT study consortium COS development group

[1] Department of Paediatric Surgery, Erasmus Medical Centre-Sophia Children's Hospital, Rotterdam, the Netherlands
[2] Department of Paediatric Surgery, Great Ormond Street Hospital, London, United Kingdom
[3] Department of Paediatric Pulmonology, Ankara University, School of Medicine, Ankara, Turkey
[4] Department of Paediatric Pulmonology, Hospital Universitari Vall d´Hebron, Barcelona, Spain
[5] Department of Paediatric Surgery, Bambino Gesù Pediatric Hospital,Rome, Italy
[6] Department of Paediatric Surgery, Karolinska Institutet, Stockholm, Sweden
[7] Department of Paediatric Surgery, University Hospital Padua, Padua, Italy
[8] Department of Paediatric Surgery, ZNA-GZA Paola Children's Hospital, Antwerp, Belgium
[9] Department of Paediatric Surgery, Medical University of Graz, Graz, Austria
[10] Department of Paediatric Surgery, Helsinki Children's Hospital, Helsinki, Finland
[11] Department of Paediatric Surgery, Radboud University Medical Centre-Amalia Children's Hospital, Nijmegen, the Netherlands
[12] Department of Paediatric Surgery, Ghent University Hospital, Ghent, Belgium
[13] Department of Paediatric Surgery, Alder Hey Children's Hospital, School Of Health and Life Science, University of Liverpool, Liverpool, United Kingdom
[14] Department of Paediatric Surgery, Oslo University Hospital, Oslo, Norway
[15] Department of Paediatric Surgery, Erasmus Medical Centre-Sophia Children's Hospital, Rotterdam, the Netherlands

Corresponding Author:
J.M. Schnater, MD, PhD
Department of Paediatric Surgery, Erasmus MC – Sophia Children's Hospital
Postal address: Postbus 2060. 3000 CB Rotterdam
Email: j.schnater@erasmusmc.nl
Telephone number: (+31)107036214

Word count: 1954 words

For peer review only

Abstract

Introduction

A worldwide lack of consensus exists on the optimal management of asymptomatic Congenital Pulmonary Airway Malformation (CPAM) even though the incidence is increasing. Either a surgical resection is performed or a wait-and-see policy is employed, depending on the treating physician. Management is largely based on expert-opinion and scientific evidence is scarce. Wide variations in outcome measures are seen between studies making comparison difficult thus highlighting the lack of universal consensus in outcome measures as well. We aim to define a core outcome set which will include the most important core outcome parameters for paediatric patients with an asymptomatic CPAM.

Methods and analysis

This study will include a critical appraisal of the current literature followed by a three-stage Delphi process with two stakeholder groups. One surgical group including paediatric as well as thoracic surgeons, and a non-surgeon group including paediatric pulmonologists, intensive care and neonatal specialists. All participants will score outcome parameters according to their level of importance and the most important parameters will be determined by consensus.

Ethics and Dissemination

Electronic informed consent will be obtained from all participants. Ethical approval is not required. After the core outcome set has been defined, we intend to design an international randomized controlled trial: The CONNECT (COllaborative Neonatal NEtwork for the first CPAM Trial),which will be aimed at determining the optimal management of asymptomatic CPAM patients.

Strength and limitations of this study

- No consensus exists on the optimal management of patients with an asymptomatic Congenital Pulmonary Airway Malformation (CPAM).
- Prospective studies on postnatal management are lacking, and cohort studies vary widely in end terms.
- Optimal timing and modality to monitor asymptomatic patients are unknown.
- Core outcome sets are a disease-specific collection of the most important outcomes that have been identified by consensus between key stakeholders.
- This protocol describes an international online Delphi survey aimed at identifying the most important core outcome parameters for paediatric patients with an asymptomatic CPAM.

Keywords: congenital lung disease, lung malformation, pediatric pulmonology, Congenital cystic adenomatoid malformation, Congenital pulmonary airway malformation

For peer review only

Introduction

Congenital Pulmonary Airway Malformation (CPAM), formerly known as Congenital Cystic Adenomatoid Malformation (CCAM), is the most common congenital lung abnormality (CLA) [1]. The incidence of CPAM has increased up to 4 per 10,000 births over the last years [2]. Most infants born with a CPAM are asymptomatic but others may show symptoms such as neonatal respiratory distress, persistent cough or recurrent lung infections in the first years of life [3]. A worldwide lack of consensus exists on the optimal management and follow-up of infants with an asymptomatic CPAM [4,5]. Prospective studies on postnatal management are lacking, and cohort studies vary widely in the outcome measures they report [2,6].

An asymptomatic CPAM is either surgically resected or a wait-and-see policy is employed depending on physician preference or local guidelines. Either way, cases are ideally discussed in a multidisciplinary team in which parental preferences are taken into account as well. To date, no definitive evidence exists on the optimal management, long-term outcomes are still unknown, and factors for predicting symptoms are still being investigated [6]. The arguments for a wait-and-see policy are that the malformation is originally benign, potentially regresses, and in most cases remains asymptomatic [7]. The arguments for surgical management include the risk of recurrent lung infections (which could make subsequent surgery more difficult), risk of acute respiratory distress, potential malignant transformation, parental anxiety, and allowing for compensatory lung growth [8,9].

Consensus needs to be reached on outcome measures and their timing that can be applied in international studies aimed at identifying the optimal management of asymptomatic CPAM. A core outcome set (COS) is a disease-specific collection of outcomes that have been identified by consensus between key stakeholders as being the most important in determining success of a treatment [10]. Such consensus is often reached through a Delphi method in which stakeholders anonymously rate outcome measures according to their importance, in one or multiple rounds [11]. We aim to develop a COS for CPAM patients using the Delphi method as a tool for reaching consensus and present the study protocol for this.

Methods

The COS development will follow the Core Outcome Set-STAndards for Development (COS-STAD) Recommendations [12] and the Core Outcome Measures in Effectiveness Trials (COMET) handbook [11]. This COS development study was registered with the COMET Initiative in May 2020 (http://www.comet-initiative.org/studies/details/1570).

Scope

This protocol describes the Delphi method which shall define a COS for all asymptomatic patients who are either prenatally or postnatally diagnosed with a CPAM. Asymptomatic patients are defined as those who have no need for prolonged respiratory support (> 24 hours) including supplemental oxygen and ventilation. The COS will include the most important outcome measures for CPAM patients, regardless of the management. The COS may be used as a guideline for clinical follow-up or in future research studies. After the COS has been defined, we intend to design a randomized controlled trial: The CONNECT (COllaborative Neonatal NEtwork for the first European CPAM Trial). This trial will be performed by the CONNECT study consortium and aims to identify the optimal management of asymptomatic CPAM patients. The COS is intended for pediatric patients up until the age of 18 years and will not include fetal outcome. Outcome parameters as well as their measurement instruments, and age at assessment will be included.

Study design

The COS will be developed in an online, three-round, Delphi process, preceded by an appraisal of previously published literature.

For peer review only

Literature review

A systematic literature review is recommended to yield an initial outcome set for the first round in the Delphi process [11]. To date, no randomized studies have yet been done and other studies examining the management of asymptomatic CPAMs report a large variety in outcome parameters [6]. A literature review will be done by the study management coordinators (SH, CK) informed by two literature reviews, previously published in a special issue of a pediatric surgery scientific journal, each covering opposing thoughts on the arguments for surgical management [8] or a wait-and-see policy [7]. In addition, we will scrutinize a recent systematic review and meta-analysis, which covers the risks associated with either surgical resection or a wait-and-see policy of asymptomatic CPAM [3]. Existing definitions, measurement tools and common measurement time-points for outcomes will be extracted , and formatted into appropriately phrased questions for use in the first round of the Delphi process. The independent coordinators will be blinded for participant identity during the process by means of a unique identification number and will ensure the Delphi process is performed according to the protocol.

Stakeholders and recruitment

Pediatric surgeons and thoracic surgeons are the health care professionals who are most frequently involved in the operative management of CPAM patients. Initial consultation or follow-up is variably done by pediatric surgeons, maternal-fetal medicine specialists, neonatologists, pediatricians or pediatric pulmonologists. We therefore decided to form two stakeholder groups: (1) Surgeons (i.e. pediatric and thoracic surgeons); and (2) Non-surgeons (e.g. maternal-fetal medicine specialists, pediatricians, pediatric pulmonologists and neonatologists).

We will recruit study participants through an existing international network of pediatric surgeons and pulmonologists who have expressed interest in collaborating on the CONNECT trial. We will inform potential participants on the aims and procedures of the Delphi process and encourage them to enroll other specialists involved in the care of CPAM patients in their own centers. Prior to the first round, those who have been found willing to participate will be sent an email explaining the aims and procedures of the Delphi process, and emphasizing the importance of finishing each round within the allocated time.

Sample size

There is no consensus on the optimal sample size for a Delphi study [11]; recruitment will therefore be based on the prospective study for which it is primarily intended (CONNECT). For the CONNECT trial, we aim to include at least 12 international centres, and therefore set the minimum of participants in each stakeholder group in the final round at 12. To reduce bias, no more than two participants from a single centre can participate in a stakeholder group. To minimize attrition bias in consecutive rounds, we aim to achieve that 75% of participants complete a round [11,13]. Therefore, the minimum number of participants in each stakeholder group for round 1 will be 21, and 16 for round 2. We believe that these minimum numbers constitute a representative sample, considering the rarity of the disease and the limited number of professionals with experience in managing this disease. Previous Delphi COS studies in paediatric surgery used similar numbers for investigating a more common disease such as appendicitis [14,15].

Attrition bias

Attrition bias will be assessed separately for each round of the Delphi process, and separately in each stakeholder group. In each group, the median score of every outcome will be compared using the Wilcoxon rank-sum test, between those only completing the previous round and those completing the consecutive round as well [11].

Delphi study

For peer review only

Consensus

Participants will be asked to score each outcome parameter using the Grading of Recommendations, Assessment, Development and Evaluations (GRADE) scale [16]. The 9-point Likert scale will label 1–3 as “not important”, 4–6 as “important but not critical” and 7–9 as “critical”. Consensus for inclusion is reached if ≥70% of participants rate the outcome parameter 7–9 and <15% rate it 1–3. Consensus for exclusion is reached if >70% participants rate the outcome 1–3 and <15% rate it 7–9. Outcomes not meeting these definitions will be classified as ‘no consensus’ [11].

Timeline

Participants will be asked to complete each round of the Delphi process within 4 weeks. A weekly reminder email will be sent to those who then have not yet completed the survey. Those failing to complete the questionnaire within the allocated 4 weeks will be excluded from next rounds. The deadline shall be extended if the projected minimum sample size has not been reached and those failing to complete the questionnaire shall be approached individually.

Delphi round 1

The three-round Delphi process shall be performed using “Welphi”, an online data system specifically developed for this use [17]. All participants shall be approached simultaneously and asked to rate each of the previously identified outcome parameters on importance as follows: *“How important would you rate the following outcome parameter including measurement instrument and age in determining the best management of asymptomatic CPAM patients? “*.

Participants are invited to suggest additional outcome parameters stating: (1) the outcome parameter, (2) the measurement instrument, and (3) the age at assessment. These additional outcome parameters will be scored in the second round.

Delphi round 1 analysis

Outcome parameters will be analyzed separately for each stakeholder group (surgeons and non-surgeons) and all parameters will be included in the second round of the Delphi process. The additional outcomes provided by participants will be reviewed to confirm they represent new outcomes. If confirmed, the item in question will be included in the second round as well.

Delphi round 2

All participants who completed the first round will automatically be invited to participate in round 2. Per stakeholder group, the median scores assigned in the first round will be made known. This will allow participants to consider the views of the other participants in the stakeholder group. They will be invited to look at all items again and consider adjusting their own scores. Furthermore, they will be asked to score the newly added outcome parameters suggested in the first round.

Delphi round 2 analysis

All outcome parameters meeting consensus criteria for exclusion by all participants will be excluded from the third round. All other parameters will be included in the third round.

Delphi round 3

All participants who completed the first and second rounds will be invited to participate in the third round. The median score of their own stakeholder group and the score of the other stakeholder group will be presented to participants. This will allow participants to consider the views of the other stakeholder group before rescoring the outcomes. They will be invited to look at all remaining items again and consider adjusting their own scores. In addition, participants will be asked to identify a single outcome parameter which is the most important for determining the treatment choice in asymptomatic CPAM patients according to them.

For peer review only

Delphi round 3 analysis and final COS development

All outcome parameters meeting the criteria for consensus of inclusion by all participants, will be included in the final core outcome set. All other outcome parameters will be excluded. To achieve a COS which is feasible for clinical use in trials, we aim to include a maximum of 10 outcome parameters in the final COS. If the number of outcome parameters meeting the criteria for consensus of inclusion greatly exceeds this maximum number, we will only include the 10 outcomes with the highest level of consensus for the COS and report those excluded in this stage. The level of consensus will be determined by the median score of each outcome parameter in round 3.
The final COS will be a collection of the most important outcome parameters in CPAM patients. The final COS will be annotated according to the outcome taxonomy which was constructed to maximize future data harmonization. Additionally, the final COS will be divided into the four core areas of the OMERACT filter: death, life impact, pathophysiological manifestations, and resource use [10].

Ethics and dissemination

Electronic informed consent will be obtained from all participants. Prior ethical approval for the Delphi study is not required. The final COS will be published in an international peer-reviewed scientific journal and on the COMET Initiative website (http://www. comet- initiative.org).

Data collection and confidentiality
Participants will complete questionnaires using the “Welphi” survey tool [17]. Anonymized data will be stored on a secure online server and will be managed according to the European General Data Protection Regulation [18].

For peer review only

Contributors
All authors contributed to the design of this protocol. SH, DM, NC, SG, PB, CMB, AS, SH, JS, MS, LD, KE, HAT, RW, JMS initiated the project. The protocol was drafted by SH and CK. The protocol was critically reviewed by DM, NM, NC, SG, PB, CMB, AS, SH, HT, JS, MS, LD, PDL, KE, HAT, RW, JMS. All authors contributed to the manuscript and read and approved the final manuscript. The CONNECT study consortium COS development group consists of all participants of the Delphi process. They have all read, refined and approved the final manuscript.

Funding statement
The authors have not received a specific grant for this research from any funding agency in the public, commercial or non-profit sectors.
Competing interests
None declared.

Patient and public involvement
No patient involved

Provenance and peer review
Not commissioned; externally peer reviewed.

Collaborators: The following people form the CONNECT study consortium COS development group: S.M. Hermelijn, C.M. Kersten, J.M. Schnater, R.M.H. Wijnen, H.A.W.M. Tiddens, S.C.M. Cochius - den Otter (Sophia Children's Hospital, Rotterdam-the Netherlands); J. Suominen, M. Pakarinen, L. Martelius (Helsinki Children's Hospital, Helsinki-Finland); S. Heyman, D. Vervloessem (ZNA-GZA Paola Children's Hospital, Antwerp-Belgium); H. Steyaert (Queen Fabiola Children's University Hospital, Brussels-Belgium); A. Sgrò, P. Gamba (University Hospital Padua, Padua-Italy); M. Schurink, S. van der Heide, J. Roukema (Amalia Children's Hospital, Nijmegen-the Netherlands); N. Rikkers-Mutsaerts (Leiden University Medical Centre, Leiden-the Netherlands); S. Terheggen-Lagro, S. de Beer, E. Haarman (Amsterdam University Medical Centre, Amsterdam-the Netherlands); H. Till, G. Singer (Medical University of Graz, Graz-Austria); M. Metzelder, P Sezen (Medizinische Universität Wien, Vienna-Austria); L. Desender, H. Schaballie (Ghent University Hospital, Ghent-Belgium); N. Cobanoglu, G. Gollu (Ankara University, School of Medicine, Ankara-Turkey); M. Stanton (University Hospital Southampton, Southampton-United Kingdom); A. Bonnard (Hopital Universitaire Robert Debré, Paris-France); R. Sfeir (Hopital Jeanne de Flandre, Lille-France); N. Muthialu, D. Mullassery, C. Wallis (Great Ormond Street Hospital, London-United Kingdom); D. Cox (Children's Health Ireland at Crumlin, Dublin-Ireland); P. Bagolan, F. Morini (Bambino Gesù Pediatric Hospital, Rome-Italy); C. Mesas Burgos, P. Conner, E. Caffrey Osvald, C. Bitkover (Karolinska Institutet, Stockholm-Sweden); H. Decaluwé, M. Proesmans, M. Boon, J. Deprest (University Hospital Leuven, Leuven-Belgium); S. Gartner, A. Lain (Hospital Universitari Vall d´Hebron, Barcelona-Spain); P.D. Losty, I. Sinha (Alder Hey Children's Hospital, School Of Health and Life Science, University of Liverpool, Liverpool-United Kingdom); I. Yardley (Evelina London Children's Hospital, London-United Kingdom); M. Singh (Birmingham Children's Hospital, Birmingham-United Kingdom); L. Wessel, K. Zahn, T. Schaible (University Hospital Mannheim, Mannheim-Germany); N. Qvist (Odense Univeristy Hospital, Odense-Denmark); M. Zampoli (Red Cross Children's Hospital, Cape town-South Africa); G. Aksnes (Oslo University Hospital, Oslo-Norway); C. K. van der Ent, K.M. Winter-de Groot (University Medical Centre Utrecht, Utrecht-the Netherlands); R. Peters (Royal Manchester Children's Hospital, Manchester-United Kingdom); E. Hannon (Leeds Children's Hospital, Leeds-United Kingdom); Q. Jöbsis, M. Bannier (Maastricht University Medical Centre, Maastricht-the Netherlands).

For peer review only

References

1. EUROCAT. Prevalence tables. 2007-2018. http://www.eurocat-network.eu/ACCESSPREVALENCEDATA/PrevalenceTables (accessed Jan 2019).
2. Stocker LJ, Wellesley DG, Stanton MP, Parasuraman R, Howe DT. The increasing incidence of foetal echogenic congenital lung malformations: an observational study. *Prenat Diagn* 2015; **35**(2): 148-53.
3. Stanton M, Njere I, Ade-Ajayi N, Patel S, Davenport M. Systematic review and meta-analysis of the postnatal management of congenital cystic lung lesions. *J Pediatr Surg* 2009; **44**(5): 1027-33.
4. Lo AY, Jones S. Lack of consensus among Canadian pediatric surgeons regarding the management of congenital cystic adenomatoid malformation of the lung. *J Pediatr Surg* 2008; **43**(5): 797-9.
5. Morini F, Zani A, Conforti A, et al. Current Management of Congenital Pulmonary Airway Malformations: A "European Pediatric Surgeons' Association" Survey. *Eur J Pediatr Surg* 2018; **28**(1): 1-5.
6. Wong KKY, Flake AW, Tibboel D, Rottier RJ, Tam PKH. Congenital pulmonary airway malformation: advances and controversies. *Lancet Child Adolesc Health* 2018; **2**(4): 290-7.
7. Stanton M. The argument for a non-operative approach to asymptomatic lung lesions. *Semin Pediatr Surg* 2015; **24**(4): 183-6.
8. Singh R, Davenport M. The argument for operative approach to asymptomatic lung lesions. *Semin Pediatr Surg* 2015; **24**(4): 187-95.
9. Hermelijn SM, Wolf JL, Dorine den Toom T, et al. Early KRAS oncogenic driver mutations in nonmucinous tissue of congenital pulmonary airway malformations as an indicator of potential malignant behavior. *Hum Pathol* 2020; **103**: 95-106.
10. Boers M, Kirwan JR, Gossec L, et al. How to choose core outcome measurement sets for clinical trials: OMERACT 11 approves filter 2.0. *J Rheumatol* 2014; **41**(5): 1025-30.
11. Williamson PR, Altman DG, Bagley H, et al. The COMET Handbook: version 1.0. *Trials* 2017; **18**(Suppl 3): 280.
12. Kirkham JJ, Davis K, Altman DG, et al. Core Outcome Set-STAndards for Development: The COS-STAD recommendations. *PLoS Med* 2017; **14**(11): e1002447.
13. Bruce I, Harman N, Williamson P, et al. The management of Otitis Media with Effusion in children with cleft palate (mOMEnt): a feasibility study and economic evaluation. *Health Technol Assess* 2015; **19**(68): 1-374.
14. Sherratt FC, Eaton S, Walker E, et al. Development of a core outcome set to determine the overall treatment success of acute uncomplicated appendicitis in children: a study protocol. *BMJ Paediatr Open* 2017; **1**(1): e000151.
15. Knaapen M, Hall NJ, van der Lee JH, et al. Establishing a core outcome set for treatment of uncomplicated appendicitis in children: study protocol for an international Delphi survey. *BMJ Open* 2019; **9**(5): e028861.
16. Guyatt GH, Oxman AD, Kunz R, et al. GRADE guidelines: 2. Framing the question and deciding on important outcomes. *J Clin Epidemiol* 2011; **64**(4): 395-400.
17. Welphi online survey platform. 2020. www.welphi.com.
18. (GDPR) Gdpr. Regulation (EU) 2016/679 of the European Parliament and of the Council of 27 April 2016 on the protection of natural persons with regard to the processing of personal data and on the free movement of such data, and repealing Directive 95/46/EC (General Data Protection Regulation), OJ 2016 L 119/1. 2018.

For peer review only

From: Core Outcome Set-STAndardised Protocol Items: the COS-STAP Statement

TITLE/ABSTRACT		
Title<br>Page 1	1a	Identify in the title that the paper describes the protocol for the planned development of a COS
Abstract<br>Page 2	1b	Provide a structured abstract
INTRODUCTION		
Background and objectives<br>Page 3	2a	Describe the background and explain the rationale for developing the COS, and identify the reasons why a COS is needed and the potential barriers to its implementation
	2b	Describe the specific objectives with reference to developing a COS
Scope<br>Page 3	3a	Describe the health condition(s) and population(s) that will be covered by the COS
	3b	Describe the intervention(s) that will be covered by the COS
	3c	Describe the context of use for which the COS is to be applied
METHODS		
Stakeholders<br>Page 4	4	Describe the stakeholder groups to be involved in the COS development process, the nature of and rationale for their involvement and also how the individuals will be identified; this should cover involvement both as members of the research team and as participants in the study
Information sources<br>Page 4	5a	Describe the information sources that will be used to identify the list of outcomes. Outline the methods or reference other protocols/papers
	5b	Describe how outcomes may be dropped/combined, with reasons
Consensus process<br>Page 5	6	Describe the plans for how the consensus process will be undertaken
Consensus definition<br>Page 5	7a	Describe the consensus definition
	7b	Describe the procedure for determining how outcomes will be added/combined/dropped from consideration during the consensus process
ANALYSIS		
Outcome scoring/feedback<br>Page 5	8	Describe how outcomes will be scored and summarised, describe how participants will receive feedback during the consensus process

For peer review only

Missing data<br>Page 5	9	Describe how missing data will be handled during the consensus process
ETHICS and DISSEMINATION		
Ethics approval/informed consent<br>Page 6	10	Describe any plans for obtaining research ethics committee/institutional review board approval in relation to the consensus process and describe how informed consent will be obtained (if relevant)
Dissemination<br>Page 6	11	Describe any plans to communicate the results to study participants and COS users, inclusive of methods and timing of dissemination
ADMINISTRATIVE INFORMATION		
Funders<br>Page 7	12	Describe sources of funding, role of funders
Conflicts of interest<br>Page 7	13	Describe any potential conflicts of interest within the study team and how they will be managed

For peer review only

BMJ Open

Development of a Core Outcome Set for Congenital Pulmonary Airway Malformations: study protocol of an international Delphi survey

Journal:	*BMJ Open*
Manuscript ID	bmjopen-2020-044544.R2
Article Type:	Protocol
Date Submitted by the Author:	17-Mar-2021
Complete List of Authors:	Hermelijn, Sergei; Erasmus MC Sophia Children Hospital, Pediatric Surgery<br>Kersten, Casper; Erasmus MC Sophia Children Hospital, Pediatric Surgery<br>Mullassery, Dhanya; Great Ormond Street Hospital for Children<br>Muthialu, Nagarajan; Great Ormond Street Hospital for Children<br>Cobanoglu, Nazan; Ankara University Faculty of Medicine<br>Gartner, Silvia; Vall d'Hebron Hospital Universitari<br>Bagolan, Pietro; Ospedale Pediatrico Bambino Gesu, Department of Medical and Surgical Neonatology<br>Mesas Burgos, Carmen; Karolinska Institutet<br>Sgro, Alberto; Padua University Hospital<br>Heyman, Stijn; ZNA-GZA Paola Children's Hospital<br>Till, Holger; Medical University of Graz<br>Suominen, Janne; University of Helsinki Children's Hospital<br>Schurink, Maarten; Radboud University Medical Centre Amalia Children's Hospital<br>Desender, Liesbeth; Ghent University Faculty of Medicine and Health Sciences<br>Losty, Paul; University of Liverpool,<br>Ertresvag, Kjetil; Oslo University Hospital<br>Tiddens, Harm A. W. M.; Erasmus MC Sophia Children Hospital, Pediatric Pulmonology<br>Wijnen, Rene; Erasmus University Medical Center - Sophia Children's Hospital, Paediatric Surgery<br>Schnater, Marco; Erasmus Medical Center, Paediatric Surgery
**Primary Subject Heading**:	Surgery
Secondary Subject Heading:	Paediatrics, Respiratory medicine, Surgery
Keywords:	Paediatric intensive & critical care < ANAESTHETICS, NEONATOLOGY, Paediatric thoracic medicine < PAEDIATRICS, PAEDIATRIC SURGERY, Paediatric thoracic surgery < PAEDIATRIC SURGERY

For peer review only - http://bmjopen.bmj.com/site/about/guidelines.xhtml

I, the Submitting Author has the right to grant and does grant on behalf of all authors of the Work (as defined in the below author licence), an exclusive licence and/or a non-exclusive licence for contributions from authors who are: i) UK Crown employees; ii) where BMJ has agreed a CC-BY licence shall apply, and/or iii) in accordance with the terms applicable for US Federal Government officers or employees acting as part of their official duties; on a worldwide, perpetual, irrevocable, royalty-free basis to BMJ Publishing Group Ltd ("BMJ") its licensees and where the relevant Journal is co-owned by BMJ to the co-owners of the Journal, to publish the Work in this journal and any other BMJ products and to exploit all rights, as set out in our licence.

The Submitting Author accepts and understands that any supply made under these terms is made by BMJ to the Submitting Author unless you are acting as an employee on behalf of your employer or a postgraduate student of an affiliated institution which is paying any applicable article publishing charge ("APC") for Open Access articles. Where the Submitting Author wishes to make the Work available on an Open Access basis (and intends to pay the relevant APC), the terms of reuse of such Open Access shall be governed by a Creative Commons licence – details of these licences and which Creative Commons licence will apply to this Work are set out in our licence referred to above.

Other than as permitted in any relevant BMJ Author's Self Archiving Policies, I confirm this Work has not been accepted for publication elsewhere, is not being considered for publication elsewhere and does not duplicate material already published. I confirm all authors consent to publication of this Work and authorise the granting of this licence.

For peer review only

Development of a Core Outcome Set for Congenital Pulmonary Airway Malformations: study protocol of an international Delphi survey

S.M. Hermelijn [1], C.M. Kersten [1], D. Mullassery [2], N. Muthialu [2], N. Cobanoglu [3], S. Gartner [4], P. Bagolan [5], C. Mesas Burgos [6], A. Sgrò [7], S. Heyman [8], H. Till [9], J. Suominen [10], M. Schurink [11], L. Desender [12], P.D. Losty [13], K. Ertresvag [14], H.A.W.M. Tiddens [15], R.M.H. Wijnen [1], J.M. Schnater [1] on behalf of the CONNECT study consortium COS development group

[1] *Department of Paediatric Surgery, Erasmus Medical Centre-Sophia Children's Hospital, Rotterdam, the Netherlands*
[2] *Department of Paediatric Surgery, Great Ormond Street Hospital, London, United Kingdom*
[3] *Department of Paediatric Pulmonology, Ankara University, School of Medicine, Ankara, Turkey*
[4] *Department of Paediatric Pulmonology, Hospital Universitari Vall d´Hebron, Barcelona, Spain*
[5] *Department of Paediatric Surgery, Bambino Gesù Pediatric Hospital,Rome, Italy*
[6] *Department of Paediatric Surgery, Karolinska Institutet, Stockholm, Sweden*
[7] *Department of Paediatric Surgery, University Hospital Padua, Padua, Italy*
[8] *Department of Paediatric Surgery, ZNA-GZA Paola Children's Hospital, Antwerp, Belgium*
[9] *Department of Paediatric Surgery, Medical University of Graz, Graz, Austria*
[10] *Department of Paediatric Surgery, Helsinki Children's Hospital, Helsinki, Finland*
[11] *Department of Paediatric Surgery, Radboud University Medical Centre-Amalia Children's Hospital, Nijmegen, the Netherlands*
[12] *Department of Paediatric Surgery, Ghent University Hospital, Ghent, Belgium*
[13] *Department of Paediatric Surgery, Alder Hey Children's Hospital, School Of Health and Life Science, University of Liverpool, Liverpool, United Kingdom*
[14] *Department of Paediatric Surgery, Oslo University Hospital, Oslo, Norway*
[15] *Department of Paediatric Surgery, Erasmus Medical Centre-Sophia Children's Hospital, Rotterdam, the Netherlands*

Corresponding Author:
J.M. Schnater, MD, PhD
Department of Paediatric Surgery, Erasmus MC – Sophia Children's Hospital
Postal address: Postbus 2060. 3000 CB Rotterdam
Email: j.schnater@erasmusmc.nl
Telephone number: (+31)107036214

Word count: 1954 words

For peer review only

Abstract

Introduction

A worldwide lack of consensus exists on the optimal management of asymptomatic Congenital Pulmonary Airway Malformation (CPAM) even though the incidence is increasing. Either a surgical resection is performed or a wait-and-see policy is employed, depending on the treating physician. Management is largely based on expert-opinion and scientific evidence is scarce. Wide variations in outcome measures are seen between studies making comparison difficult thus highlighting the lack of universal consensus in outcome measures as well. We aim to define a core outcome set which will include the most important core outcome parameters for paediatric patients with an asymptomatic CPAM.

Methods and analysis

This study will include a critical appraisal of the current literature followed by a three-stage Delphi process with two stakeholder groups. One surgical group including paediatric as well as thoracic surgeons, and a non-surgeon group including paediatric pulmonologists, intensive care and neonatal specialists. All participants will score outcome parameters according to their level of importance and the most important parameters will be determined by consensus.

Ethics and Dissemination

Electronic informed consent will be obtained from all participants. Ethical approval is not required. After the core outcome set has been defined, we intend to design an international randomized controlled trial: The CONNECT (COllaborative Neonatal NEtwork for the first CPAM Trial),which will be aimed at determining the optimal management of asymptomatic CPAM patients.

Strength and limitations of this study

- The core outcome set is a disease-specific collection of the most important outcomes that will be established by consensus between key stakeholders.
- Participants will be an international group of specialists with experience in the treatment of CPAM patients which will result in a universally approved synthesis of expert-opinion.
- This protocol describes an international online Delphi survey that should identify the most important core outcome parameters including optimal timing and modality to monitor paediatric patients with an asymptomatic CPAM.
- Existing comprehensive literature reviews shall be used to inform initial outcome parameters instead of a systematic literature search as no randomized trials and very few prospective studies have yet been published regarding the outcome of CPAM patients.
- Parent and patients' views are not included in the protocol as the final core outcome set is specifically intended for asymptomatic CPAM patients, however their input will play an important role when designing future studies.

Keywords: congenital lung disease, lung malformation, pediatric pulmonology, Congenital cystic adenomatoid malformation, Congenital pulmonary airway malformation

For peer review only

Introduction

Congenital Pulmonary Airway Malformation (CPAM), formerly known as Congenital Cystic Adenomatoid Malformation (CCAM), is the most common congenital lung abnormality (CLA) [1]. The incidence of CPAM has increased up to 4 per 10,000 births over the last years [2]. Most infants born with a CPAM are asymptomatic but others may show symptoms such as neonatal respiratory distress, persistent cough or recurrent lung infections in the first years of life [3]. A worldwide lack of consensus exists on the optimal management and follow-up of infants with an asymptomatic CPAM [4,5]. Prospective studies on postnatal management are lacking, and cohort studies vary widely in the outcome measures they report [2,6].

An asymptomatic CPAM is either surgically resected or a wait-and-see policy is employed depending on physician preference or local guidelines. Either way, cases are ideally discussed in a multidisciplinary team in which parental preferences are taken into account as well. To date, no definitive evidence exists on the optimal management, long-term outcomes are still unknown, and factors for predicting symptoms are still being investigated [6]. The arguments for a wait-and-see policy are that the malformation is originally benign, potentially regresses, and in most cases remains asymptomatic [7]. The arguments for surgical management include the risk of recurrent lung infections (which could make subsequent surgery more difficult), risk of acute respiratory distress, potential malignant transformation, parental anxiety, and allowing for compensatory lung growth [8,9].

Consensus needs to be reached on outcome measures and their timing that can be applied in international studies aimed at identifying the optimal management of asymptomatic CPAM. A core outcome set (COS) is a disease-specific collection of outcomes that have been identified by consensus between key stakeholders as being the most important in determining success of a treatment [10]. Such consensus is often reached through a Delphi method in which stakeholders anonymously rate outcome measures according to their importance, in one or multiple rounds [11]. We aim to develop a COS for CPAM patients using the Delphi method as a tool for reaching consensus and present the study protocol for this.

Methods

The COS development will follow the Core Outcome Set-STAndards for Development (COS-STAD) Recommendations [12] and the Core Outcome Measures in Effectiveness Trials (COMET) handbook [11]. This COS development study was registered with the COMET Initiative in May 2020 (http://www.comet-initiative.org/studies/details/1570).

Scope

This protocol describes the Delphi method which shall define a COS for all asymptomatic patients who are either prenatally or postnatally diagnosed with a CPAM. Asymptomatic patients are defined as those who have no need for prolonged respiratory support (> 24 hours) including supplemental oxygen and ventilation. The COS will include the most important outcome measures for CPAM patients, regardless of the management. The COS may be used as a guideline for clinical follow-up or in future research studies. After the COS has been defined, we intend to design a randomized controlled trial: The CONNECT (COllaborative Neonatal NEtwork for the first European CPAM Trial). This trial will be performed by the CONNECT study consortium and aims to identify the optimal management of asymptomatic CPAM patients. The COS is intended for pediatric patients up until the age of 18 years and will not include fetal outcome. Outcome parameters as well as their measurement instruments, and age at assessment will be included.

Study design

The COS will be developed in an online, three-round, Delphi process, preceded by an appraisal of previously published literature.

For peer review only

Literature review
A systematic literature review is recommended to yield an initial outcome set for the first round in the Delphi process [11]. To date, no randomized studies have yet been done and other studies examining the management of asymptomatic CPAMs report a large variety in outcome parameters [6]. A literature review will be done by the study management coordinators (SH, CK) informed by two literature reviews, previously published in a special issue of a pediatric surgery scientific journal, each covering opposing thoughts on the arguments for surgical management [8] or a wait-and-see policy [7]. In addition, we will scrutinize a recent systematic review and meta-analysis, which covers the risks associated with either surgical resection or a wait-and-see policy of asymptomatic CPAM [3]. Existing definitions, measurement tools and common measurement time-points for outcomes will be extracted , and formatted into appropriately phrased questions for use in the first round of the Delphi process. The independent coordinators will be blinded for participant identity during the process by means of a unique identification number and will ensure the Delphi process is performed according to the protocol.

Stakeholders and recruitment
Pediatric surgeons and thoracic surgeons are the health care professionals who are most frequently involved in the operative management of CPAM patients. Initial consultation or follow-up is variably done by pediatric surgeons, maternal-fetal medicine specialists, neonatologists, pediatricians or pediatric pulmonologists. We therefore decided to form two stakeholder groups: (1) Surgeons (i.e. pediatric and thoracic surgeons); and (2) Non-surgeons (e.g. maternal-fetal medicine specialists, pediatricians, pediatric pulmonologists and neonatologists).
We will recruit study participants through an existing international network of pediatric surgeons and pulmonologists who have expressed interest in collaborating on the CONNECT trial. We will inform potential participants on the aims and procedures of the Delphi process and encourage them to enroll other specialists involved in the care of CPAM patients in their own centers. Prior to the first round, those who have been found willing to participate will be sent an email explaining the aims and procedures of the Delphi process, and emphasizing the importance of finishing each round within the allocated time.

Sample size
There is no consensus on the optimal sample size for a Delphi study [11]; recruitment will therefore be based on the prospective study for which it is primarily intended (CONNECT). For the CONNECT trial, we aim to include at least 12 international centres, and therefore set the minimum of participants in each stakeholder group in the final round at 12. To reduce bias, no more than two participants from a single centre can participate in a stakeholder group. To minimize attrition bias in consecutive rounds, we aim to achieve that 75% of participants complete a round [11,13]. Therefore, the minimum number of participants in each stakeholder group for round 1 will be 21, and 16 for round 2. We believe that these minimum numbers constitute a representative sample, considering the rarity of the disease and the limited number of professionals with experience in managing this disease. Previous Delphi COS studies in paediatric surgery used similar numbers for investigating a more common disease such as appendicitis [14,15].

Attrition bias
Attrition bias will be assessed separately for each round of the Delphi process, and separately in each stakeholder group. In each group, the median score of every outcome will be compared using the Wilcoxon rank-sum test, between those only completing the previous round and those completing the consecutive round as well [11].

Delphi study

For peer review only

Consensus
Participants will be asked to score each outcome parameter using the Grading of Recommendations, Assessment, Development and Evaluations (GRADE) scale [16]. The 9-point Likert scale will label 1–3 as "not important", 4–6 as "important but not critical" and 7–9 as "critical". Consensus for inclusion is reached if ≥70% of participants rate the outcome parameter 7–9 and <15% rate it 1–3. Consensus for exclusion is reached if >70% participants rate the outcome 1–3 and <15% rate it 7–9. Outcomes not meeting these definitions will be classified as 'no consensus' [11].

Timeline
Participants will be asked to complete each round of the Delphi process within 4 weeks. A weekly reminder email will be sent to those who then have not yet completed the survey. Those failing to complete the questionnaire within the allocated 4 weeks will be excluded from next rounds. The deadline shall be extended if the projected minimum sample size has not been reached and those failing to complete the questionnaire shall be approached individually.

Delphi round 1
The three-round Delphi process shall be performed using "Welphi", an online data system specifically developed for this use [17]. All participants shall be approached simultaneously and asked to rate each of the previously identified outcome parameters on importance as follows: *"How important would you rate the following outcome parameter including measurement instrument and age in determining the best management of asymptomatic CPAM patients? "*.
Participants are invited to suggest additional outcome parameters stating: (1) the outcome parameter, (2) the measurement instrument, and (3) the age at assessment. These additional outcome parameters will be scored in the second round.

Delphi round 1 analysis
Outcome parameters will be analyzed separately for each stakeholder group (surgeons and non-surgeons) and all parameters will be included in the second round of the Delphi process. The additional outcomes provided by participants will be reviewed to confirm they represent new outcomes. If confirmed, the item in question will be included in the second round as well.

Delphi round 2
All participants who completed the first round will automatically be invited to participate in round 2. Per stakeholder group, the median scores assigned in the first round will be made known. This will allow participants to consider the views of the other participants in the stakeholder group. They will be invited to look at all items again and consider adjusting their own scores. Furthermore, they will be asked to score the newly added outcome parameters suggested in the first round.

Delphi round 2 analysis
All outcome parameters meeting consensus criteria for exclusion by all participants will be excluded from the third round. All other parameters will be included in the third round.

Delphi round 3
All participants who completed the first and second rounds will be invited to participate in the third round. The median score of their own stakeholder group and the score of the other stakeholder group will be presented to participants. This will allow participants to consider the views of the other stakeholder group before rescoring the outcomes. They will be invited to look at all remaining items again and consider adjusting their own scores. In addition, participants will be asked to identify a single outcome parameter which is the most important for determining the treatment choice in asymptomatic CPAM patients according to them.

For peer review only

Delphi round 3 analysis and final COS development

All outcome parameters meeting the criteria for consensus of inclusion by all participants, will be included in the final core outcome set. All other outcome parameters will be excluded. To achieve a COS which is feasible for clinical use in trials, we aim to include a maximum of 10 outcome parameters in the final COS. If the number of outcome parameters meeting the criteria for consensus of inclusion greatly exceeds this maximum number, we will only include the 10 outcomes with the highest level of consensus for the COS and report those excluded in this stage. The level of consensus will be determined by the median score of each outcome parameter in round 3.
The final COS will be a collection of the most important outcome parameters in CPAM patients. The final COS will be annotated according to the outcome taxonomy which was constructed to maximize future data harmonization. Additionally, the final COS will be divided into the four core areas of the OMERACT filter: death, life impact, pathophysiological manifestations, and resource use [10].

Ethics and dissemination

Electronic informed consent will be obtained from all participants. Prior ethical approval for the Delphi study is not required. The final COS will be published in an international peer-reviewed scientific journal and on the COMET Initiative website (http://www. comet- initiative.org).

Data collection and confidentiality
Participants will complete questionnaires using the "Welphi" survey tool [17]. Anonymized data will be stored on a secure online server and will be managed according to the European General Data Protection Regulation [18].

For peer review only

Contributors

All authors contributed to the design of this protocol. SH, DM, NC, SG, PB, CMB, AS, SH, JS, MS, LD, KE, HAT, RW, JMS initiated the project. The protocol was drafted by SH and CK. The protocol was critically reviewed by DM, NM, NC, SG, PB, CMB, AS, SH, HT, JS, MS, LD, PDL, KE, HAT, RW, JMS. All authors contributed to the manuscript and read and approved the final manuscript. The CONNECT study consortium COS development group consists of all participants of the Delphi process. They have all read, refined and approved the final manuscript.

Funding statement

The authors have not received a specific grant for this research from any funding agency in the public, commercial or non-profit sectors.

Competing interests

None declared.

Patient and public involvement

No patient involved

Provenance and peer review

Not commissioned; externally peer reviewed.

Collaborators: The following people form the CONNECT study consortium COS development group: S.M. Hermelijn, C.M. Kersten, J.M. Schnater, R.M.H. Wijnen, H.A.W.M. Tiddens, S.C.M. Cochius - den Otter (Sophia Children's Hospital, Rotterdam-the Netherlands); J. Suominen, M. Pakarinen, L. Martelius (Helsinki Children's Hospital, Helsinki-Finland); S. Heyman, D. Vervloessem (ZNA-GZA Paola Children's Hospital, Antwerp-Belgium); H. Steyaert (Queen Fabiola Children's University Hospital, Brussels-Belgium); A. Sgrò, P. Gamba (University Hospital Padua, Padua-Italy); M. Schurink, S. van der Heide, J. Roukema (Amalia Children's Hospital, Nijmegen-the Netherlands); N. Rikkers-Mutsaerts (Leiden University Medical Centre, Leiden-the Netherlands); S. Terheggen-Lagro, S. de Beer, E. Haarman (Amsterdam University Medical Centre, Amsterdam-the Netherlands); H. Till, G. Singer (Medical University of Graz, Graz-Austria); M. Metzelder, P Sezen (Medizinische Universität Wien, Vienna-Austria); L. Desender, H. Schaballie (Ghent University Hospital, Ghent-Belgium); N. Cobanoglu, G. Gollu (Ankara University, School of Medicine, Ankara-Turkey); M. Stanton (University Hospital Southampton, Southampton-United Kingdom); A. Bonnard (Hopital Universitaire Robert Debré, Paris-France); R. Sfeir (Hopital Jeanne de Flandre, Lille-France); N. Muthialu, D. Mullassery, C. Wallis (Great Ormond Street Hospital, London-United Kingdom); D. Cox (Children's Health Ireland at Crumlin, Dublin-Ireland); P. Bagolan, F. Morini (Bambino Gesù Pediatric Hospital, Rome-Italy); C. Mesas Burgos, P. Conner, E. Caffrey Osvald, C. Bitkover (Karolinska Institutet, Stockholm-Sweden); H. Decaluwé, M. Proesmans, M. Boon, J. Deprest (University Hospital Leuven, Leuven-Belgium); S. Gartner, A. Lain (Hospital Universitari Vall d´Hebron, Barcelona-Spain); P.D. Losty, I. Sinha (Alder Hey Children's Hospital, School Of Health and Life Science, University of Liverpool, Liverpool-United Kingdom); I. Yardley (Evelina London Children's Hospital, London-United Kingdom); M. Singh (Birmingham Children's Hospital, Birmingham-United Kingdom); L. Wessel, K. Zahn, T. Schaible (University Hospital Mannheim, Mannheim-Germany); N. Qvist (Odense Univeristy Hospital, Odense-Denmark); M. Zampoli (Red Cross Children's Hospital, Cape town-South Africa); G. Aksnes (Oslo University Hospital, Oslo-Norway); C. K. van der Ent, K.M. Winter-de Groot (University Medical Centre Utrecht, Utrecht-the Netherlands); R. Peters (Royal Manchester Children's Hospital, Manchester-United Kingdom); E. Hannon (Leeds Children's Hospital, Leeds-United Kingdom); Q. Jöbsis, M. Bannier (Maastricht University Medical Centre, Maastricht-the Netherlands).

For peer review only

References

1. EUROCAT. Prevalence tables. 2007-2018. http://www.eurocat-network.eu/ACCESSPREVALENCEDATA/PrevalenceTables (accessed Jan 2019).
2. Stocker LJ, Wellesley DG, Stanton MP, Parasuraman R, Howe DT. The increasing incidence of foetal echogenic congenital lung malformations: an observational study. *Prenat Diagn* 2015; **35**(2): 148-53.
3. Stanton M, Njere I, Ade-Ajayi N, Patel S, Davenport M. Systematic review and meta-analysis of the postnatal management of congenital cystic lung lesions. *J Pediatr Surg* 2009; **44**(5): 1027-33.
4. Lo AY, Jones S. Lack of consensus among Canadian pediatric surgeons regarding the management of congenital cystic adenomatoid malformation of the lung. *J Pediatr Surg* 2008; **43**(5): 797-9.
5. Morini F, Zani A, Conforti A, et al. Current Management of Congenital Pulmonary Airway Malformations: A "European Pediatric Surgeons' Association" Survey. *Eur J Pediatr Surg* 2018; **28**(1): 1-5.
6. Wong KKY, Flake AW, Tibboel D, Rottier RJ, Tam PKH. Congenital pulmonary airway malformation: advances and controversies. *Lancet Child Adolesc Health* 2018; **2**(4): 290-7.
7. Stanton M. The argument for a non-operative approach to asymptomatic lung lesions. *Semin Pediatr Surg* 2015; **24**(4): 183-6.
8. Singh R, Davenport M. The argument for operative approach to asymptomatic lung lesions. *Semin Pediatr Surg* 2015; **24**(4): 187-95.
9. Hermelijn SM, Wolf JL, Dorine den Toom T, et al. Early KRAS oncogenic driver mutations in nonmucinous tissue of congenital pulmonary airway malformations as an indicator of potential malignant behavior. *Hum Pathol* 2020; **103**: 95-106.
10. Boers M, Kirwan JR, Gossec L, et al. How to choose core outcome measurement sets for clinical trials: OMERACT 11 approves filter 2.0. *J Rheumatol* 2014; **41**(5): 1025-30.
11. Williamson PR, Altman DG, Bagley H, et al. The COMET Handbook: version 1.0. *Trials* 2017; **18**(Suppl 3): 280.
12. Kirkham JJ, Davis K, Altman DG, et al. Core Outcome Set-STAndards for Development: The COS-STAD recommendations. *PLoS Med* 2017; **14**(11): e1002447.
13. Bruce I, Harman N, Williamson P, et al. The management of Otitis Media with Effusion in children with cleft palate (mOMEnt): a feasibility study and economic evaluation. *Health Technol Assess* 2015; **19**(68): 1-374.
14. Sherratt FC, Eaton S, Walker E, et al. Development of a core outcome set to determine the overall treatment success of acute uncomplicated appendicitis in children: a study protocol. *BMJ Paediatr Open* 2017; **1**(1): e000151.
15. Knaapen M, Hall NJ, van der Lee JH, et al. Establishing a core outcome set for treatment of uncomplicated appendicitis in children: study protocol for an international Delphi survey. *BMJ Open* 2019; **9**(5): e028861.
16. Guyatt GH, Oxman AD, Kunz R, et al. GRADE guidelines: 2. Framing the question and deciding on important outcomes. *J Clin Epidemiol* 2011; **64**(4): 395-400.
17. Welphi online survey platform. 2020. www.welphi.com.
18. (GDPR) Gdpr. Regulation (EU) 2016/679 of the European Parliament and of the Council of 27 April 2016 on the protection of natural persons with regard to the processing of personal data and on the free movement of such data, and repealing Directive 95/46/EC (General Data Protection Regulation), OJ 2016 L 119/1. 2018.

For peer review only

From: Core Outcome Set-STAndardised Protocol Items: the COS-STAP Statement

TITLE/ABSTRACT		
Title<br>Page 1	1a	Identify in the title that the paper describes the protocol for the planned development of a COS
Abstract<br>Page 2	1b	Provide a structured abstract
INTRODUCTION		
Background and objectives<br>Page 3	2a	Describe the background and explain the rationale for developing the COS, and identify the reasons why a COS is needed and the potential barriers to its implementation
	2b	Describe the specific objectives with reference to developing a COS
Scope<br>Page 3	3a	Describe the health condition(s) and population(s) that will be covered by the COS
	3b	Describe the intervention(s) that will be covered by the COS
	3c	Describe the context of use for which the COS is to be applied
METHODS		
Stakeholders<br>Page 4	4	Describe the stakeholder groups to be involved in the COS development process, the nature of and rationale for their involvement and also how the individuals will be identified; this should cover involvement both as members of the research team and as participants in the study
Information sources<br>Page 4	5a	Describe the information sources that will be used to identify the list of outcomes. Outline the methods or reference other protocols/papers
	5b	Describe how outcomes may be dropped/combined, with reasons
Consensus process<br>Page 5	6	Describe the plans for how the consensus process will be undertaken
Consensus definition<br>Page 5	7a	Describe the consensus definition
	7b	Describe the procedure for determining how outcomes will be added/combined/dropped from consideration during the consensus process
ANALYSIS		
Outcome scoring/feedback<br>Page 5	8	Describe how outcomes will be scored and summarised, describe how participants will receive feedback during the consensus process

For peer review only

Missing data<br>Page 5	9	Describe how missing data will be handled during the consensus process
ETHICS and DISSEMINATION		
Ethics approval/informed consent<br>Page 6	10	Describe any plans for obtaining research ethics committee/institutional review board approval in relation to the consensus process and describe how informed consent will be obtained (if relevant)
Dissemination<br>Page 6	11	Describe any plans to communicate the results to study participants and COS users, inclusive of methods and timing of dissemination
ADMINISTRATIVE INFORMATION		
Funders<br>Page 7	12	Describe sources of funding, role of funders
Conflicts of interest<br>Page 7	13	Describe any potential conflicts of interest within the study team and how they will be managed

For peer review only
